# Supplementary material for: Effects of announcing a vocabulary test before reading a glossed text on reading behaviors and vocabulary acquisition: An eye-tracking study
Source: PLoS One. 2023 Jan 19;18(1):e0280552. doi: 10.1371/journal.pone.0280552 (PMC9851552; doi:10.1371/journal.pone.0280552)
Supplement: S1 File — (DOCX) [file pone.0280552.s001.docx]

**<APPENDIX I>**

**Reading Comprehension Test**

* 질문을 읽고 알맞은 답을 기입하세요. (주관식 문항은 한글/영어 모두 가능)

1. 소년들은 무엇을 보고 겨울이 올 것이라 예상했는가? ( )

① 날씨 ② 범람한 호수 ③ 많은 새 ④ 사냥꾼의 개

1. Hudson이 몰래 빌려온 것은? ( )

① 총 ② 요리기구 ③ 사냥 옷 ④ 가방

1. 소년들이 가졌던 총알의 개수는? ( )

① 2개 ② 3개 ③ 4개 ④ 5개

1. 소년들이 새를 쏜 직후 느꼈던 감정은? ( )

① 기쁨 ② 두려움 ③ 부끄러움 ④ 슬픔

1. 소년들이 묻었던 새를 다시 보러 간 계절은 언제였는가? ( )

① 봄 ② 여름 ③ 가을 ④ 겨울

1. 소년들 중 새의 무덤을 보러 가자고 처음 이야기를 꺼낸 사람은? ( )

① me ② Hudson ③ Peter ④ Clement

1. 소년들이 새 팔이 Ali를 찾아간 이유는? ( )

① 자신들이 잡은 새의 가치를 알아보기 위해

② 새 요리 방법을 물어보기 위해

③ 자신들이 잡은 새를 잠시 숨겨달라고 부탁하기 위해

④ 사냥꾼들에게 팔기 위해

1. 사냥꾼들이 새 파는 사람들에게 새를 사는 이유는 무엇인가? ( )

① 더 싼 값에 새를 살 수 있기 때문에

② 다양한 종류의 새들을 수집하기 위해

③ 한 마리도 못 잡았을 때 사람들의 비웃음을 피하기 위해

④ 사냥개의 먹잇감으로 줄 새를 얻기 위해

1. 소년들이 무덤을 확인하러 가서 새가 밖으로 나와 있는 것을 보고 생각한 것은? ( )

① 누군가가 와서 무덤을 팠다고 생각했다

② Crane 새가 자신의 죽은 짝을 보기 위해 왔다고 생각했다

③ 무덤을 깊게 파지 않아 강한 설풍때문에 헝클어졌다고 생각했다

④ 들개가 와서 먹으려다 얼어서 먹지 못했다고 생각했다

1. 이야기의 마지막 부분에서 Crane 새들이 소년들의 집에 날아와 두고 간 꽃의 이름은?

( )

1. Crane 사건 이후 소년들의 일상에서 변화된 행동은? ( )
2. 사냥을 하지 않으려고 노력한다
3. 새들의 무덤에 꽃이 시들지 않도록 가꾼다
4. 사랑이라는 단어를 들을 때마다 새들을 생각한다
5. 주변 사람들에게 자신이 겪은 일을 이야기해주며 사냥을 주의하도록 한다
6. 사냥꾼들이 화를 내며 소년들을 쫓아왔던 이유는 무엇이었는가?

_____________________________________________________________________

1. Crane 새가 ‘사랑의 상징(symbols of love)’이라는 별명이 붙은 이유는 무엇일까요?

____________________________________________________________________

**<APPENDIX II>**

**Form Recall Test**

* 지문에서 화면 아래에 어휘와 뜻이 제시되었습니다. 그 중 생각나는 어휘가 있다면 적어주세요.

| 단어 | 뜻 |
| --- | --- |
| 1. |  |
| 2. |  |
| 3. |  |
| 4. |  |
| 5. |  |
| 6. |  |
| 7. |  |
| 8. |  |
| 9. |  |
| 10. |  |
| 11. |  |
| 12. |  |
| 13. |  |
| 14. |  |
| 15. |  |
| 16. |  |

**< APPENDIX III>**

**Meaning Recall Test**

* 다음 어휘들의 뜻을 우리말로 적어주세요.

| 단어 | 의미 |
| --- | --- |
| 1. yeel |  |
| 2. dizz |  |
| 3. tarb |  |
| 4. marve |  |
| 5. gras |  |
| 6. zerk |  |
| 7. breaply |  |
| 8. novely |  |
| 9. spoy |  |
| 10. hule |  |
| 11. vapsely |  |
| 12. fusk |  |
| 13. drine |  |
| 14. pess |  |
| 15. tately |  |
| 16. smow |  |

**<APPENDIX IV>**

**Meaning Recognition Test**

* 주어진 어휘들의 의미로 적절한 것을 고르시오

1. dizz

① 겨울 ② 추운 ③ 얼다 ④ 딱딱한 ⑤ 모르겠음

2. tately

① 후에 ② 바쁘게 ③ 전에 ④ 순식간에 ⑤ 모르겠음

3. yeel

① 큰 ② 긴 ③ 슬픈 ④ 위험한 ⑤ 모르겠음

4. spoy

① 화난 ② 무서운 ③ 흥분한 ④ 혼내다 ⑤ 모르겠음

5. tarb

① 주머니 ② 딱딱한 ③ 냄비 ④ 자루 ⑤ 모르겠음

6. hule

① 가득한 ② 멋진 ③ 다양한 ④ 형형색색의 ⑤ 모르겠음

7. marve

① 웅장한 ② 계절 ③ 봄 ④ 시작 ⑤ 모르겠음

8. drine

① 오늘 ② 다음에 ③ 내일 ④ 곧 ⑤ 모르겠음

9. zerk

① 굽다 ② 만들다 ③ 요리하다 ④ 망가트리다 ⑤ 모르겠음

10. fusk

① 움직임 ② 소음 ③ 연기 ④ 가방 ⑤ 모르겠음

11. gras

① 이해하다 ② 팔다 ③ 잡다 ④ 생각하다 ⑤ 모르겠음

12. smow

① 깊게 ② 조금씩 ③ 파다 ④ 삼키다 ⑤ 모르겠음

13. novely

① 겸손히 ② 천천히 ③ 흥미롭게 ④ 조용히 ⑤ 모르겠음

14. pess

① 지나치다 ② 생각하다 ③ 연구하다 ④ 예상하다 ⑤ 모르겠음

15. breaply

① 어느 날 ② 매우 ③ 짧게 ④ 갑자기 ⑤ 모르겠음

16. vapsely

① 빨리 ② 오래 ③ 종종 ④ 느리게 ⑤ 모르겠음
