# Supplementary material for: Effects of announcing a vocabulary test before reading a glossed text on reading behaviors and vocabulary acquisition: An eye-tracking study
Source: PLoS One. 2023 Jan 19;18(1):e0280552. doi: 10.1371/journal.pone.0280552 (PMC9851552; doi:10.1371/journal.pone.0280552)
Supplement: Attachment — Submitted filename: PONE.docx [file pone.0280552.s002.docx]

This is an interesting study that explored how test announcement prior to reading would moderate the impact of glossing on learning of form and meaning of target words. The authors employed eye-tracking technology to measure the amount of attention driven to the in-text as well as text-margin target words, and their glosses. The learning was assessed in terms of form and meaning recognition and recall abilities. The results revealed that test announcement did not have significant impact on reading comprehension, but promoted attention to the text-margin target words and form recall scores.

While this study certainly points out a meaningful gap in the literature and provides pedagogical insights on how to better utilize glosses in L2 reading instruction, there are issues that should be addressed to be considered for publication in the journal. More detailed comments are provided below.

**p. 3**

Before explaining incidental and intentional learning here, I would recommend authors to briefly review and clarify the relationship between implicit/explicit language learning and incidental/intentional language learning.

Plus, it is not that we can simply equate incidental learning as implicit learning. For instance, we do sometimes look up in a dictionary to search a meaning of an unknown word, while reading for pleasure with no liabilities.

“However, during incidental vocabulary learning, frequent exposure to

language input may take a long time to be connected to the acquisition of the vocabulary, the

wrong meaning of words may be learned, if the learner is not provided with correct cues to

help retrieve the meanings of unknown words from context.”

- citation please.

**p. 4**

This section reviews only a few glossing studies in the context of incidental learning.

- Hulstijn et al., 1996

- Ko, 2012

- Yanagisawa et al., 2020 (meta-analysis)

- Kang et al., 2020

- Choi, 2016

Why is that? There is an extensive amount of research on the impact of glossing on incidental vocabulary learning, and I'm curious why the authors reviewed only these studies..

Plus, given that one of the purposes of this study was to prove if glossing indeed promotes attention to the glossed words (as well as the glosses), they would want to review more eye-tracking studies on glossing (that is, under the no-test-announcement condition, not just Choi (2016) and Kang t al. (2020).

In the first paragraph of 2.2, Please provide more detailed explanation on the cognitive/ pedagogical mechanism in which glosses can promote incidental vocabulary learning.

**p. 5**

**2.3**

This section reads like a patchwork, loosely listing the studies without a clear logical sequence. I would recommend the authors to make this section more organic and logical, demonstrating how they synthesize the methodological aspects and the findings of the reviewed studies for readers.

Many studies have quantified the glossing effect in *incidental* vocabulary learning, whereas

relatively fewer studies seem to be done on the effect of glosses in the context of *intentional*

vocabulary learning (cf. Ko, 1995; Montero Perez et al., 2018; Peters, 2007; Peters et al., 2009; Zuo, 2021).

- Why do the authors think is the case?

**p. 6-7**

Another a bit awkward transition.. The authors should make this section more condensed, synthesized, and logical. (e.g., Further, Furthermore, Afterwards, ….) This issue pertains to the manuscript as a whole, and in particular this section (pp. 6-7).

**p. 9**

**4.3.1.**

This is good information on the reading text, but please additionally add the lexical coverage (2K and 3K?) as well as readability index.

The Turkish names of the characters were changed to English names to make it easier for participants to read. For example, ‘Hikmet’ was replaced by ‘Hudson’, and ‘Halil’ was replaced by ‘Morgan’.

- Please add explanation for this with proper citations

**4.3.2.**

The reason that pseudowords were used as the target words is that the subject's prior vocabulary knowledge may affect the experimental results.

- Add citation please.

More importantly, why were these particular words chosen? Please provide the conditions. For instance, why didn’t the authors control the part of speech of the target words? What about their task-essentialness (that is, were they important in successful reading comprehension)?

**p. 10**

**4.3.4.**

Why not form recognition? Given this was an input-based learning activity, I'd like to know why the authors did not measure form recognition.

In the form recall test, what prompt was given? Were the participants provided with meanings? Or just 16 blanks? Or were the blanks provided within meaningful context? Please provide a sample item to help readers' understanding.

**4.3.5**

Please add more detailed information on the RC questions, not just their formats. For example, what dimensions of RC was measured? Main idea/ detailed information/ true-not true informaation/ finding synonyms/ etc. Also, what was the rationale for including two different types of RC questions? More importantly, were they allowed to refer back to the reading text while answering the questions? Or they had to rely on their memory? Why?

What was the reliability? I would recommend the authors to share the text and the RC questions as appendix or supplementary item.

**p. 11**

**4.5.**

Without detailed information on the short-answer RC questions, this scoring method seems not very useful.

**p. 12**

**4.6.**

Please add information on how the raw eye-movement data were trimmed before conducting statistical analysis. For example, how were the outliers treated/ filtered out? Also, what statistical anslysis was used? Please provide detailed information on how the pre-processed eye-movement data were analyzed. Also, how were the different reading times across participants handled? This section lack too much important information that is necessary to replicate this study, so I would suggest extensive revision here.

**5.1.1.**

*d* > 0.001 ??? 🡪 *d* < 0.001

**p. 13**

“An independent-samples *t*-test was performed to analyze whether the time…”

- This exact wording is too repetitive.

Results 🡪 The results

**p. 15**

The 2^nd^ and the 3^rd^ paragraphs are very repetitive and redundant. The authors would want to focus on interpreting the results in this section.

Plus, there are studies that used eye-tracking technology to examine if glossing works.

Were the target words necessary for comprehension? The authors would want to consider the task-essentialness of the target words. That is, if the target words didn’t matter (especially adjectives or adverbs), they could have felt only little need (Laufer and Hulstijn’s Involvement Load Hypothesis) to know the meaning of the target words.

**p. 16**

The difference between these results and ours might have occurred because recall and recognition (i.e., of forms) involve different cognitive efforts.

- Please elaborate more on this.

Our results reveal that vocabulary test announcement can affect which type of vocabulary

knowledge can be acquired.

- I'm not sure if I can understand this part. The authors may want to revise it.

That is, test announcement can help the students to encode forms of new words, but that additional cognitive resources will be required to map forms to meanings of encoded words, or to consolidate form-meaning association of the encoded words.

- Glossing in and of itself, promotes learning of word meanings. Previous studies overall showed that glossing promotes learning of word meanings, rather than forms. To be more specific, when glossing is not provided, readers tend to inspect the text more intensively to infer the meaning of the unknown word, especially if the word is important in comprehension. When glossing is provided, they do not feel the need to search for the meaning, as the gloss is right there, and hence have no or even negative impact on learning word forms. Perhaps additional demands induced by the TA could have compensated this relatively weak/inhibitory impact of glossing on learning of word forms. The authors should review more studies on the impact of glossing on learning of word forms, and not to mention, Laufer and Hulstijn's Involvement Load Theory. w

We presented fruitful findings on and implications of test announcement prior to reading a

glossed text in vocabulary acquisition.

- The authors would want to revise this sentence, especially the word choice "Fruitful."

Information about the type of vocabulary test might have changed the type of vocabulary that the participants learned.

- I am not very suer if the word "type" is recommendable. The authors would want to reconsider this term throughout the manuscript to enhance clarity.

The authors would want to use the past tense when reporting what they DID or FOUND in this study throughout the methodology, results, and discussion sections.
