# Supplementary material for: Effects of announcing a vocabulary test before reading a glossed text on reading behaviors and vocabulary acquisition: An eye-tracking study
Source: PLoS One. 2023 Jan 19;18(1):e0280552. doi: 10.1371/journal.pone.0280552 (PMC9851552; doi:10.1371/journal.pone.0280552)
Supplement: Attachment — Submitted filename: Review Comments.docx [file pone.0280552.s003.docx]

Comments on “Effects of vocabulary test announcement prior to reading a glossed text on reading behaviors and vocabulary acquisition: An eye movement study”

The paper is in general well written. The research was carefully designed and the use of eye-tracking method is innovative. There are a number of issues, however, that need to be considered by the researchers.

1. Concerning form / meaning distinction

It is good that the researchers, based on previous studies, went a step further to measure the effect of test announcement on form recall and meaning recall. The researchers, however, did no review literature about the form / meaning distinction in L2 vocabulary learning. Since form / meaning are key constructs in the research, there need to be more discussions about them in the literature review.

2. In the research, there are three ROIs. What are the reasons of setting three ROIs for each target word? Please state the rationale.

Isn’t the bottom margin-target word a part of the gloss?

3. Why are there F2 words and F4 words? The frequency of the target words is not a variable in this research.

4. What are the purposes of carrying out a meaning recall test and a meaning recognition test? Please justify this.

5. Was the form recall test more like a memory test instead of a vocabulary learning test?

6. The scoring method for the meaning recall test can be improved since it seemed to be more flexible compared with the form recall test. The researchers are encouraged to try: 0 – incorrect; 1 – partially correct; 2 – correct.

7. Another issue is about the reading comprehension questions. Are they related to the target words? Explanations are needed in the manuscript.
8. Are the target words important for the comprehension of the text? This is to be explained in the research design.
9. Test announcement is one step to initiate L2 learners’ noticing of the target word in reading. Whether the words are retained or not depends largely on the learner’s subsequent processing of the words, esp. depth of processing. As the researchers reviewed in the manuscript, a number of studies have been done on the effect of test announcement on L2 vocabulary learning, it is suggested that in future research the researchers focus more on processing depth and the mechanisms leading to differential learning outcomes of word form / meaning.
