# Supplementary material for: Effects of announcing a vocabulary test before reading a glossed text on reading behaviors and vocabulary acquisition: An eye-tracking study
Source: PLoS One. 2023 Jan 19;18(1):e0280552. doi: 10.1371/journal.pone.0280552 (PMC9851552; doi:10.1371/journal.pone.0280552)
Supplement: Attachment — Submitted filename: Review Comments.docx [file pone.0280552.s004.docx]

Comments on “Effects of vocabulary test announcement prior to reading a glossed text on reading behaviors and vocabulary acquisition: An eye movement study”

The paper is in general well written. The research was carefully designed and the use of eye-tracking method is innovative. There are a number of issues, however, that need to be considered by the researchers.

1. Concerning form / meaning distinction

It is good that the researchers, based on previous studies, went a step further to measure the effect of test announcement on form recall and meaning recall. The researchers, however, did no review literature about the form / meaning distinction in L2 vocabulary learning. Since form / meaning are key constructs in the research, there need to be more discussions about them in the literature review.

Response:

We added the explanation of form-meaning distinction in L2 vocabulary learning as below in the revised manuscript.

“The in-text target words are presented in the text and the bottom-margin target words are occurred at the bottom of the text in L2. Both correspond to the ‘form’ of a word which can also be called morphological information such as spelling or pronunciation (Jiang, 2000). When learners look at the in-text or bottom-margin target words, they may acquire the orthographic lexical form of the words (i.e., fusk, tarb). On the other hand, glosses enable learners to obtain semantic information, that is the ‘meaning’ of words (i.e., 소음, 가방). It may encourage learners to activate the form-meaning links (i.e., fusk-소음, tarb-가방). Thus, the reading time that the participants spent gazing at the ‘in-text target words’ and ‘bottom-margin target words’ can be related to the acquisition of ‘form’ of words, and the time spent reading the glosses can be related to the ‘form-meaning connection’ of words.”

2. In the research, there are three ROIs. What are the reasons of setting three ROIs for each target word? Please state the rationale.

Response:

We added the following in the revised manuscript.

“We set an ROI for each of three words: *in-text* target word, *bottom-margin* target word, and gloss, respectively.”

Please understand that we did NOT set three ROIs for each target word. Rather, we set an ROI for each of three words (1) In-text target word, (2) bottom-margin target word, and (3) Gloss, respectively.

Isn’t the bottom margin-target word a part of the gloss?

Response:

We defined a gloss as a definition or short explanation of an unfamiliar word in a text. Thus, we differentiated a bottom-margin target word (i.e., hule) from a L1 gloss (i.e., 가득찬). Also, when a participant looks at a gloss, she looks for the meaning of the target word, not its form. Thus, we set different ROIs for a bottom-margin target word and a gloss. Of course, a target word should be presented with its meaning (i.e., gloss), otherwise, we do not know of which word a gloss is. Mostly, glosses are presented this way in textbooks and many reading materials.

3. Why are there F2 words and F4 words? The frequency of the target words is not a variable in this research.

Response:

As we first started the current research, we wanted to see the frequency effects of the word as well. However, we determined not to see the frequency effects, because the conditions of the words of two frequency groups were not controlled in terms of different contexts and distributions.

4. What are the purposes of carrying out a meaning recall test and a meaning recognition test? Please justify this.

Response:

We added the following in the revised manuscript.

“We used two different form-meaning association tests to examine whether test announcement can affect the mapping of form to meaning differently according to different tests. More specifically, the meaning recall test measures relatively stronger form-meaning association by asking to recall a meaning of a given target word, rather than the meaning recognition test that allows to choose the best answer among multiple-choices.”

5. Was the form recall test more like a memory test instead of a vocabulary learning test?

Response:

Yes, it was. Please understand that learning new words basically entails memory of novel word forms (Jiang, 2000). Previous research on vocabulary acquisition conducted the meaning recall test as we did.

6. The scoring method for the meaning recall test can be improved since it seemed to be more flexible compared with the form recall test. The researchers are encouraged to try: 0 – incorrect; 1 – partially correct; 2 – correct.

Response:

We understand your concern. However, please understand that most glossing studies utilized the same scoring methods as in our study. Basically, we considered form-meaning association was successfully done when a participant provided a synonym of the target word. Thus, we scored a partially correct answer (synonym) as correct answer (the same word). Please understand that this binominal method of scoring did not hinder examining participants’ acquisition of meaning of words.

7. Another issue is about the reading comprehension questions. Are they related to the target words? Explanations are needed in the manuscript.

Response:

We added the following in the revised manuscript.

“In the reading comprehension test, some questions were related to the target words. For example, in order to answer a particular question asking which character did a specific behavior in which season, a participant needed to remember the form and meaning of a target word ‘marve’ meaning ‘봄’ (*spring* in Korean) presented as an option out of four choices. For a reading comprehension test item in which ‘marve’ is an answer, see question 5 in Appendix I.”

8. Are the target words important for the comprehension of the text? This is to be explained in the research design.

Response:

We added the following in the revised manuscript.

“Both sets of target words consisted of four nouns, four verbs, four adjectives, all of which were content words. The fact that all target words were content words might have facilitated participants’ accurate and fast comprehension of the text. However, it seems to be impossible to objectively measure to what extent they are important.”

9. Test announcement is one step to initiate L2 learners’ noticing of the target word in reading. Whether the words are retained or not depends largely on the learner’s subsequent processing of the words, esp. depth of processing. As the researchers reviewed in the manuscript, a number of studies have been done on the effect of test announcement on L2 vocabulary learning, it is suggested that in future research the researchers focus more on processing depth and the mechanisms leading to differential learning outcomes of word form / meaning.

Response:

We added the following in the revised manuscript. Thank you for the suggestion.

“As a reviewer suggested, further research should focus on L2 learners’ depth of processing and the mechanisms leading to differential learning outcomes of word form and meaning to see the effect of test announcement”
